# Lessons learnt from the MAGNET Malawian-German Hospital Partnership: the German perspective on contributions to patient care and capacity development

**DOI:** 10.1186/s12992-017-0270-4

**Published:** 2017-07-26

**Authors:** Florian Neuhann, Sandra Barteit

**Affiliations:** University Hospital Heidelberg, Institute of Public Health, Heidelberg, Germany

**Keywords:** Hospitals, Teaching (MeSH [D006784]), Developing Countries (MeSH [I01.615.500.300]), Malawi (MeSH [Z01.058.290.175.500]), Hospitals, District (MeSH [N02.278.421.510.140]), Germany (MeSH [Z01.542.315]), Hospitals, University (MeSH [N02.278.020.300.310]), Global Health (MeSH [H02.403.371]), Public Health (MeSH [H02.403.720]), Capacity Building (MeSH [N02.138, N04.452.105]), Primary Health Care (MeSH [N04.590.233.727])

## Abstract

**Background:**

Malawi is a low-income country with one of the highest HIV prevalence rates worldwide (Kendig et al., Trop Med Health 41:163–170, 2013). The health system depends largely on external funding. Official German development aid has supported health care in Malawi for many years (German Embassy Lilongwe, The German Development Cooperation in Malawi), including placing medical doctors in various departments of the Kamuzu Central Hospital (KCH) in Lilongwe. In 2008, a hospital partnership called MAGNET (Malawi German Networking for Capacity Building in Treatment, Training and Research at KCH) evolved as part of the German ESTHER network. The partnership was abruptly terminated in 2015.

**Methods:**

We reviewed 35 partnership documents and conducted an online survey of partnership stakeholders to retrospectively assess the hospital partnership based on the Capacity WORKS model of the German Corporation for International Cooperation (GIZ). This model evaluates systems’ management and implementation to understand and support the functioning of cooperation within societies. Based on this model, we considered the five success factors for cooperation management: (1) strategy, (2) cooperation, (3) steering, (4) processes, and (5) learning and innovation. In an online survey, we used an adapted version of the partnership evaluation tool by the Centers for Disease Control and Prevention (CDC).

**Results:**

From 2008 to 2015, the MAGNET partnership contributed to capacity building and improved patient care in the KCH Medical Department through clinical care, technical support, teaching and trainings, and operations research based on mutually agreed upon objectives. The MAGNET partnership was implemented in three phases during which there were changes in leadership in the Medical Department and the hospital, contractual policies, funder priorities and the competing influences of other actors. Communication and follow up among partners worked best during phases when a German doctor was onsite. The partnership was judged as a positive driver for change and support within the Medical Department, but eventually failed to implement self-sustainable, robust processes within the partnership to cope with multiple changes and challenges.

**Conclusion:**

The MAGNET partnership made a considerable contribution to patient care, continuous medical education and operations research at KCH, despite its abrupt termination. Changes in the institutional infrastructure, donor policy and interpersonal relations contributed to the loss of shared expectations and the end of the project.

Institutional-hospital partnerships, like MAGNET, can make a valuable contribution to health care provision and hence a wider health agenda, provided there is a flexible, mutually agreed upon strategy, personal commitment, continuous communication and robust processes. However, partnership projects remain vulnerable to the influences of external actors and structures. Ministries of Health and donor agencies should appreciate the particular strength of hospital partnerships.

## Background

In resource-limited settings, there are manifold ways of partnering and supporting health services. These include initiatives of private individual doctors or nurses, small associations, faith-based (between parishes) organisations, non-governmental organisations (NGOs) and fully institutionalized and funded partnership programs [1]. The position statement of the Global Catalyst Group (GCG) for Institutional Health Partnerships highlighted that hospital and institutional partnerships are essential for capacity strengthening and achieving global health targets and development goals [2, 3].

In 2008, based on more than a decade of collaboration through the German Development Cooperation [4], a hospital partnership was established between the KCH Medical Department and a consortium of three German university clinics: Heidelberg (Public Health, Tropical Medicine), Cologne (Infectious Diseases) and Bonn (Infectious Diseases from 2008 to 2012). The partnership, coined Malawi German Networking for Capacity Building in Treatment, Training and Research (MAGNET), was embedded in the European Hospital Network named *Ensemble pour une solidarité thérapeutique hospitalière en réseau* (ESTHER) [5]. MAGNET’s primary focus was on interventions for capacity strengthening, improvement of clinical service delivery and operational research.

ESTHER’s objective, initiating hospital partnerships, was to improve health outcomes in low- and middle-income countries, especially with regards to the diagnosis and treatment of HIV and AIDS. Members of ESTHER found that partnerships could “be very effective in addressing other health challenges […] with both sides benefitting from the two-way learning experience” [6]. The focus is on “knowledge transfer through reciprocal exchange visits, training on the job in German and African hospitals and regular joint monitoring of progress” [3, 6]. However, the contribution and effectiveness of hospital partnerships have rarely been assessed. In this paper, we describe the history of one partnership, evaluate the outputs and outcomes including the project’s eventual failure, and lessons learnt.

### Setting

#### Malawi

Malawi is one of the poorest countries of the world. The integrated household survey 2010–2011, showed that 50,7% of the 14,2 million population lived below the national poverty line [7]. Malawi is highly affected by the HIV epidemic [8, 9]. Health indicators still show high rates of infant mortality (71/1.000 live births) [10], maternal mortality (634/100.000), and tuberculosis (Tb) (227/100.000) [9, 11]. The population grows at 3,32% per year, which is a number unmatched by an adequate increase in health infrastructure and hospital services [12, 13]. Furthermore, Malawi faces a severe shortage in all health personnel reflected in the physician population ratio of 2/100.000 [9]. The situation has been aggravated by political and economic crises, such as the 2011 fuel crisis [14] and the 2013 governmental embezzlement scandal that halted international donor support [15]. The crises profoundly impacted public services including one of the largest hospitals, the Kamuzu Central Hospital (KCH) in Lilongwe. There were delays in salary payments, lack of supplies (e.g. drugs and laboratory reagents), and a consequential negative impact on staff morale [16–18].

#### Kamuzu Central Hospital

In 1977, the KCH opened as a public tertiary health care facility providing all major medical services and fulfilling a three-fold function: (1) a referral hospital for central Malawi with a growing catchment population of approximately six million, (2) a teaching and training institution for the College of Medicine (CoM), Health Sciences and Nursing, and (3) a central hospital for supervisory and mentoring visits to district hospitals in Malawi’s central region [19].

Services at the KCH are free of charge by referral at the point of delivery [19]. In 2014, the bed capacity was 1200 [19] including 114 beds in the Medical Department [20]. The Medical Department holds daily outpatient clinics and an admitting ward and is responsible for the off-campus Tb ward and psychiatric wards. Outpatient clinics exist for general medicine, diabetes, hypertension and chronic renal disease. Patients present with all medical conditions including neurological. HIV/AIDS-related conditions were predominant at the beginning of 2000 and still play a major role [20] since almost every other adult medical patient is HIV-positive at KCH [8, 21].

The Medical Department has never been adequately staffed to provide medical care and teaching. Between 2002 and 2012, one or no Malawian medical specialist was available for clinical and managerial tasks as head of department (HoD). Clinical officers (CO), clinical officer interns and medical doctor (MD) interns were responsible for day-to-day care management with one or two registrars (doctors with advanced trainings). The number of these cadres varied over time. There were two to three permanent senior COs. During an average rotation, there could be between two to four MD interns and another two to four CO interns. When the CoM’s Lilongwe Campus opened in 2012, third-year medical students from the CoM Lilongwe campus joined the KCH for their clinical rotation and more registrars were assigned to the Medical Department. However, the number of nurses remained insufficient, meaning that nurses occasionally had to work two consecutive shifts or only one nurse covered the night shift for a ward.German medical specialists have worked regularly in the Medical Department under the Integrated Expert Programme by the Centre for International Migration and Development (CIM) [22] since the late 1990s. Over time, the department also received doctors from other external partners from the UK, US, Egypt (UN Volunteers) and China. There was no formal process of coordination between the hospital administration and external partners with regards to the provision of doctors with various specializations coming to KCH. Supportive services like diagnostic radiology, clinical laboratory and pharmacy were hampered by the lack of resources and irregular supplies. A pathology lab was not available until July 2011 [23].

## Methods

### Document review

We analysed 35 documents covering the MAGNET partnership period from 2008 to 2015 including: project proposals, log frames, meeting minutes, evaluations, publications and reports (work plan meeting, project visit, annual project, external monitoring and annual meeting reports). The framework for analysis is based on the success factors of the Capacity WORKS (CW) model [24] covering: strategy, cooperation, steering structure, processes, learning and innovation. CW is a model specifically targeting cooperation management within sustainable development environments. CW covers aspects of programme design, implementation, internal evaluation and reporting and provides a structured approach for multi-stakeholder dialogues and understanding the complexity of cooperation. We selected and adapted guiding questions of the CW model to identify the success factors that were most applicable to the ESTHER-MAGNET partnership (see Table 1).

**Table 1:** Selected Guiding Questions from the CW model chosen for the evaluation of the ESTHER-MAGNET partnership

Success factors of CW model	Selected guiding questions
Strategy	▪ What is the mutually agreed and defined common goal? ▪ Which strategic options to reach the goal? ▪ How can the partnership make use of strengths? ▪ What can the partnership contribute to alleviate weaknesses? ▪ What opportunities and energy for change is available?
Cooperation	▪ Who are the relevant actors? ▪ Which mandate, roles and interests have the stakeholders? ▪ Are resources sufficient to reach the objectives? ▪ How to deal with conflicts and asymmetries of power within the cooperation system? ▪ Which comparative advantages make the cooperation system to an attractive partner?
Steering	▪ Are there measurable indicators for decision in steering of the partnership? ▪ What would be an appropriate monitoring system? ▪ How are decisions for resource allocation negotiated, agreed and implemented? ▪ Is there an operational plan for the strategic concept? ▪ How can the steering structure be modelled to enrich the cooperative culture?
Processes	▪ Which are the relevant processes in the area of activity (hospital) and how are they organized? ▪ How is the relation between central processes for performance, cooperation learning, steering and support and what are strengths and weaknesses? ▪ Can the change processes serve as model solutions?
Learning and Innovation	▪ Are there explicit learning objectives in the project? ▪ Which are the learning needs with regard to various levels of human capacity development? ▪ Is there competence in the partnership to develop sustainable cooperation, decision making and processes? ▪ How can it be assured that learning occurs from the concrete activities in the partnership? ▪ How does the partnership support continuous learning processes by various mechanisms (selection, variation, stabilization)? ▪ How are learning experiences prepared and documented?

To assess human capacity development (HCD) we follow the definition used by GIZ, which highlights the support and the shaping of individual learning processes and networking of people [25].

### Survey

We conducted an online survey among MAGNET partnership stakeholders, medical staff, partner institutions and funding agents. Responses were anonymous and covered the professional role and the time the respondent had been involved in the partnership. The survey was conducted as part of the ESTHER-MAGNET partnership and was completely voluntary, thus ethical approval was not required. We did not collect identifying information such as names, email addresses or IP addresses. Information was aggregated so no individual survey could be associated with specific responses. The survey was based on the partnership evaluation by Centers for Disease Control and Prevention [26] and included 39 statements rated on a 5-point Likert scale from “Strongly Disagree, Disagree, Neutral, Agree, Strongly Agree”. The survey was conducted from mid-November 2015 through December 2015. In total, four batches of survey invitations were sent to 32 email addresses. The survey covered 39 statements that were part of six overall partnership themes: partnership environment, membership characteristics, process and structure, communication, purpose and resources. The answers were coded. Means and standard deviations were calculated and are presented as overall ratings according to the country of the respondent.

## Results

### Limitations

As a reflection of the final disruption of the partnership, this analysis is authored only by representatives from the lead German partner institution, thereby limiting the perspective on the partnership. However, we have carefully looked at all pertaining documents and used established tools for the evaluation to reduce the bias in judgement. Despite a survey return rate of 65%, the results may be biased by respondent self-selection, thus the most critical views toward the partnership may be missing.

### Document review

Thirty-five available documents pertaining to the partnership serve as the basis to reconstruct the history and analyze the partnership with regards to the five success factors of cooperation management according to the Capacity Works model and the three partnership periods from 2008 to 2010, from 2010 to 2012, and from 2013 to 2015.

#### History of the partnership

Following the work of a single German doctor at KCH from 2002 and 2004, official German support expanded through personal networking. From 2005 to 2008, members of the Institute of Public Health of Heidelberg University and the Infectious Disease Clinics of the University Clinics of Bonn and Cologne sent two registrars during their medical specialization to the KCH Medical Department for one year (partially supported by CIM). The German registrars provided clinical care and taught local clinical officers and interns, while at the same time they learnt about the clinical presentation of infectious diseases, diagnosis and treatment with limited resources, and conducted small operational research projects. After Germany joined the European ESTHER Alliance in 2004 [5], the MAGNET partnership was initiated in 2008 with annual funding ranging between €50.000–100.000 for three project periods: 2008–2010, 2010–2012 and 2013–2015. The MAGNET partnership added a new level of potential for developing a peer-to-peer relationship to address knowledge and capacity gaps with an emphasis on empowerment and leadership [3].

##### 2008–2010

In the first MAGNET phase, emphasis was placed on support and facilitation of teaching and training, operations research on priority issues, mentorship of medical interns and the placement of additional medical doctors to support clinical care, in particular for HIV-related conditions. The aim was to develop and implement clinical protocols considering local needs, as well as national and international standards of common medical conditions. The purpose was to support on-call patient management by interns, junior doctors and clinicians without direct senior support. The protocols for treatment and management were uniformly structured to cover diagnosis, first necessary examinations and initiation of treatment for conditions such as pneumonia, cardiac failure, cryptoccocal meningitis, hypertension, stroke, renal failure, asthma, diabetic ketoacidosis and hyperosmolar non-ketotic state, liver cirrhosis, headache, carbamate poisoning, sepsis and epilepsy. The German doctor also supported the regular capacity-building activities to be implemented at KCH with German partner universities. Other plans included the development of an IT infrastructure to introduce tele-teaching and e-learning [27] and to strengthen department administration. An analysis of KCH’s existing HIV/AIDS workplace programme was conducted to determine how to improve hospital staff safety, include staff from district hospitals in capacity-strengthening activities, consolidate partnership structure and processes, and establish professional exchange on both national and international levels.

##### 2010–2012

Within ESTHER, funding priorities for the second period moved towards reproductive health. The initial MAGNET objectives were continued, but added three additional objectives: (1) improving the quality of diagnostic standards and management of febrile illnesses, (2) conducting operational research and auditing priority issues, and (3) implementing regular capacity-building activities on both hospital and district levels, and in German partner institutions. The main strategic components for this period included re-establishing microbiological diagnostics in the lab, sustaining basic haematological diagnoses, expanding ultrasonography capacity, reviewing use of anti-infective drugs and conducting a study on the causes of fever in patients admitted with febrile conditions. The hospital director decided to create an Antibiotic Stewardship (ABS) committee to expand the initiative beyond the Medical Department. Furthermore, a computer lab was established in the Medical Department with four computers to improve access to updated medical information via online resources and also as a base for a medical e-learning component that was introduced to alleviate the lack of senior medical teachers in the department, as part of MAGNET [27].

##### 2013–2015

At the end of the second MAGNET phase, an external review was commissioned by the GIZ country health program to support future funding decisions. Some review recommendations were integrated into the project proposal for the third partnership period. The focus on improving the clinical and managerial capacity of the department was continued with specific objectives on infectious diseases and non-communicable disease (NCD) management. For infectious diseases, the department was to use the hospital-wide clinical microbiology service and play an active role in the ABS initiative. For NCDs, an analysis of the patient population of the diabetes clinic was conducted. To enhance departmental management, a conducive environment was to be established for management and teaching. A logbook for medical intern rotations was introduced and interns benefited from blended learning with a medical e-learning platform [27]. For each of the objectives, the head of the department appointed a department registrar to act as the local coordinator of their field of interest. The involvement of a local coordinator worked well for the medical e-learning platform with a committed registrar [27]. For the diabetes clinic, the appointed registrar was only partly active, while the registrar appointed for infectious disease showed no engagement.

#### Strategy

For all project periods, the hospital partnership worked on mutually agreed goals aligned with the Malawian National Strategies [28–30] and the GIZ Malawian German Health Programme [31] within the administration of the Medical Department. The major objective of the partnership was to strengthen clinical care, managerial capacity and in-service training in the Medical Department. Priority was set on intern supervision and mentoring to alleviate the lack of local senior doctors for medical teaching and training. Another key component was strengthening operational research to collect local data for relevant health problems and protocols, as well as to prepare appropriate health-technology transfer. Cooperation with other locally active international initiatives within the Medical Department were sought particularly with the University of Pittsburgh’s visiting resident programme [32] and the University of North Carolina’s UNC-Lilongwe Malawi project [33]. Overall, the partnership goals (see Table 2) were broadly defined, in particular during the first partnership phase.

**Table 2:** Overall ESTHER-MAGNET partnership goals for all three periods of the partnership

Time period	Direct outcomes of the partnership
2008–2010	– Capacity-building through improved training of medical staff of the KCH Medical Department – Improved patient care in the KCH Medical Department and associated District Hospitals with a focus on HIV/AIDS and related conditions – Operational research in HIV/AIDS and related conditions – Exchange of expert knowledge between KCH Medical Department and University Hospitals of Bonn/Cologne/Heidelberg
2010–2012	– Improvement of quality of patient care and in service training of interns at the KCH Medical Department ▪ The proportion of patients who undergo appropriate diagnosis and management procedures for febrile illness is increased ▪ Blended learning including tele-teaching session are established during training at the KCH Medical Department
2013–2015	– Clinical care and managerial capacity at the KCH Medical Department are improved and the department increasingly fulfils its role as a centre for tertiary care for Malawi’s central region ▪ An overall improvement in diagnosis and management procedures for patients with infectious diseases and diabetes is enhanced in the KCH Medical Department ▪ An improved and well- functioning Medical Department management structure is in place thus achieving medical and teaching objectives and utilising the e-learning platform [27]

#### Cooperation

The two most relevant actors for the MAGNET partnership through all three partnership phases were the German partner institutions and the KCH Medical Department, and explicitly their active representatives. Other actors included the hospital administration and indirectly the Ministry of Health (MoH), the ESTHER GIZ secretariat, the GIZ country health programme, other KCH departments and several external partners, e.g. the UNC-Lilongwe Project, the University of Pittsburgh Residents Program for Health Care for the Underserved Populations and Dundee University programme for medical students. The MAGNET partner institutions and the Medical Department had little to no influence on other stakeholder decisions even when they were relevant for the partnership.

The partnership objectives were focussed on issues mainly controlled by the KCH Medical Department and reflected the limited financial resources for personnel. The partnership aimed to foster an incentivised work environment by participating in operational studies, introducing annual departmental meetings appreciating accomplished work, providing access to up-to-date and quality medical information, and participating in continuing medical education. Within operational research projects, such as a fever study [34], the partnership supported the laboratory with supplies like reagents for routine diagnostics and a new microscope, as well as stationery for the medical wards. From 2009 onwards, a small operational budget was available and handled by the KCH accounts department, which reported quarterly to the local GIZ office. The management of this account proved challenging throughout the third MAGNET period and eventually failed. One reason was that accountants expected extra pay for managing this project budget, since they argued that officially they were really only responsible for governmental accounts.

During the overall MAGNET partnership period, the KCH had five hospital directors who were principally supportive of the partnership, but not equally involved. The director during the second period attended a quality management course at the partner university, as did a department matron. However, the MoH assigned the director to another position and the matron left for parental leave. Likewise, the position of the HoD changed five times during the partnership. This high turnover of local partnership representatives compared to the continuity of the German partners posed a challenge to cooperation. The resulting need to re-define the roles and responsibilities of the Malawian partners was equally challenging and created an imbalance in the partnership.

From 2009 to 2012, the partnership allocated two German registrars to the KCH Medical Department who were vital in fortifying the link between the Malawian and the German institutions, and facilitating regular information flow. In 2010, the contract of the current German specialist in the Medical Department ended without replacement and the government placed the Malawian specialist elsewhere, thus leaving the department without established leadership. The result was that an overburdened junior German registrar was left in charge. This changed only when an experienced German medical specialist was placed at both the CoM and the Medical Department through another GIZ programme. Regular communication was based on email exchange and mobile phone calls between the HoD, the German project coordinator and the onsite German registrar. The annual departmental meetings were central to discuss the work plan for the coming year. The partnership tried to establish cooperation with the CoM by exploring ways to merge the respective e-learning platforms.

For the third period, additional funds had been mobilized for the ABS and a memorandum of understanding was signed between the donor, KCH, Heidelberg University and a local trust for financial management. During the initial meeting, there was disagreement on the previously agreed upon management of human resources for the ABS initiative. The launch was aborted, and no alternative procedure was suggested by KCH, culminating in the end of the initiative. In spring 2015, the ESTHER German officer negatively assessed the partnership leading to disruption in communication between the German and Malawian partners and further straining cooperation within the partnership. An assessment was part of each MAGNET partnership period since each period had specific objectives and was evaluated by an ESTHER German officer (or externally) before continuation. In October 2015, the partnership not renewed at the end of the third contract period.

#### Steering

Steering functions can be allocated to three main drivers: the partners, the funding agency and external actors. Negotiations and agreements among partners - mainly between the German coordinator and the Malawian HoD - were the primary steering components. Phase-specific aims and objectives were developed in cooperation with the acting HoD in consultation with the hospital director, the matron of the Medical Department, the laboratory and German representatives of the University Clinics. Actions to reach the strategic aims were reviewed in regular visits, trainings and teaching ward rounds, and in routine internal meetings. Strategic adjustments during the three distinct periods were discussed, agreed upon and forwarded to GIZ for endorsement.

Processes during the first phase were loosely formalised between the German main partner institution and the GIZ ESTHER secretariat. Funding was based on a contract between one German partner university and the ESTHER secretariat, and included the agreement of all partners. Initially, funds included a 25% full-time equivalent (FTE) for partnership coordination and administration. By 2010, the contractual framework was formalized and the GIZ country office had to approve changes in strategy, log frame and budget. The approval process took almost three months during which no expenditure was possible. Funding was no longer available for staff. Steering processes and funds allocation were also influenced by other actors, e.g., the MoH accepted a donation of ten dialysis machines during the second partnership period. The identified space for the extended dialysis unit was part of the Medical Department, which was reconstructed and led to the medical wards being spread over three floors, and the newly equipped computer lab became inaccessible. Construction work rendered technical equipment inoperable. During the second period, the partners agreed to re-introduce gastro-enterological endoscopy, however, this did not occur since it was decided to integrate and support the dialysis unit.

In general, progress was informally reviewed during regular partner visits. Since 2011, reviews were extended to annual departmental meetings and official monitoring visits by ESTHER Germany in preparation for the annual departmental meetings. These meetings developed as a vital event for providing dedicated time to identify and discuss issues of the department and the partnership.

By the end of the second project phase, external consultants commissioned by the GIZ country health programme evaluated the partnership without prior notification of the partners. The evaluation determined the continuation of GIZ support. The evaluation concluded that there had been reasonable project outputs in light of relatively little input, and timely implementation. The suggestion was to reduce and simplify activities based on clear, realistic log-frame indicators, a structured work plan, and a country-led project management. It was recommended to shift the focus towards registrar capacity-building through CoM, and to ensure follow up on activities initiated in the second phase, and notably to translate the fever study results into practice.

#### Processes

Partnership processes - such as regular communication, reporting, financial accounting, annual meetings - had to be implemented alongside existing structures and processes within the KCH and the Medical Department. A situational analysis for establishing a health care quality-improvement process in the department noted that many departmental processes were informal and subject to change depending on circumstances such as availability of staff and supplies [20]. The partnership stimulated the introduction of new processes such as minutes of departmental meetings to facilitate the identification and discussion of departmental issues, mortality reports, afternoon handover meetings to improve patient management during after hours care, and logbooks for intern rotations to structure their medical training and evaluation. Also, the intern logbook commenced to support the teaching, learning and supervision of interns, as well as the medical e-learning platform [27], which complemented the intern rotation with qualitative up-to-date materials specifically tailored to the needs of medical interns and eased information access to online resources. In the first half of 2014, a cross-sectional analysis of patients registered at the diabetes clinic was conducted introducing processes to improve diabetes management [25].

The presence of a German registrar served as an important mechanism to support the introduction and sustainment of these processes, since working from a log frame and reporting were new and foreign to many members of the partnership. Within the department, explicit planning was limited or rendered obsolete by management changes, shortages in personnel and finances or interfering decisions by other actors. For example, another GIZ project with the CoM was initiated through the German Malawian Health program, without incorporating the GIZ funded ESTHER-MAGNET partnership. Yet, the new cooperation acted as a strengthening factor for the partnership, since a German medical specialist functioned as the coordinator for the third-year medical students and also worked in the Medical Department ward.

#### Learning and innovation

Human capacity development through learning and innovation was a central aspect of the MAGNET partnership. This included the development of personal competencies, institutional learning and partnership adaptation to changing environments. Examples are operational research to improve patient care, medical e-learning [27] for capacity building, management standards of common conditions, intern logbooks for structured medical rotations and opportunities for attending courses and conferences. Given the shortage of senior doctors, intern mentoring was central to the partnership. During this crucial training phase between being a student and becoming a fully responsible medical doctor or clinical officer, partnership support included access to up-to-date information, treatment protocols to guide interns and exchanges with the partnership’s medical professionals. A local clinical officer coordinated the e-learning computer lab [27] which eventually led to the opportunity to upgrade his education to a Bachelor’s degree in Medicine.

An annual meeting played an important role in institutional learning. All departmental units prepared reports about their achievements and challenges throughout the year. Accomplishments and shortcomings were appreciated, and ownership of the work and the department was acknowledged and discussed.

The partnership fostered field studies by Master Students for International Health. This lead to four Master theses supporting operational research and strengthening of personal competence and learning. Local training courses were held in the use of Epi Info software [35], ultrasonography, and the development of departmental treatment guidelines. For undergraduates, MAGNET facilitated medical elective visits from German students, and supported the Scottish elective programme. Senior staff of the department attended conferences in Germany, visited German university hospitals, and participated in short courses in international health in Germany.

Dissemination of research findings on local and international levels provided an opportunity for human capacity development. For example, a workshop was organised with the Malawian Malaria programme and the Medical Association of Malawi for the study of febrile illnesses. To date, MAGNET has produced four peer-reviewed publications (see Table 3).

**Table 3:**
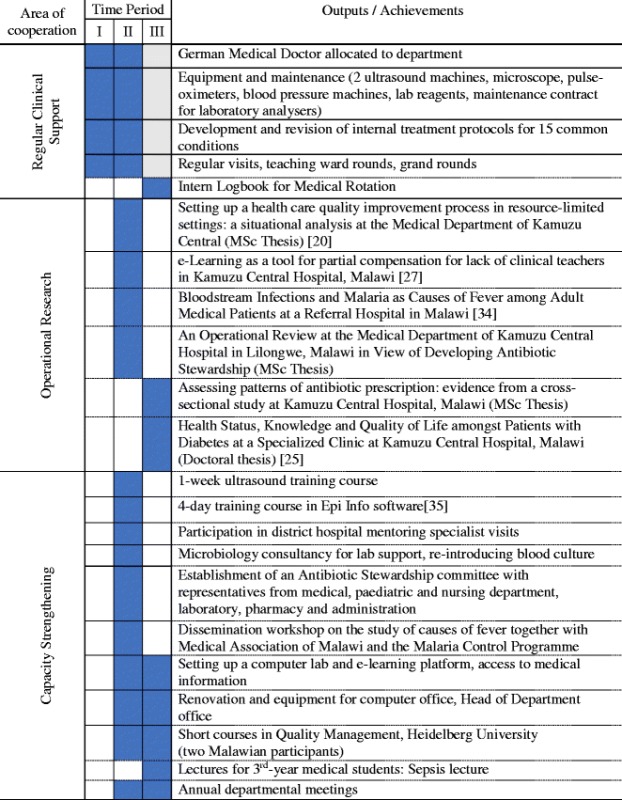
Overview of ESTHER-MAGNET partnership achievements over three partnership periods

### Survey

Overall, 21 individual responses from 11 Malawian and 10 German partners were collected using a survey. The professional roles were as follows: 12 medical doctors, 1 lab technician, 3 consultants, 2 pharmacists, 1 research assistant, 1 nurse supervisor and 1 project donor representative. Most respondents were actively involved in the partnership after 2010, with a peak involvement from 2012 to 2014.

The overall assessment showed relatively low ratings for resources, processes and structure and membership characteristics. The skilled leadership of the partnership and the establishment of informal relationships and communication skills during the partnership received the highest ratings.

Results showed that there was a positive attitude towards the history of collaboration.

**Fig. 1:**
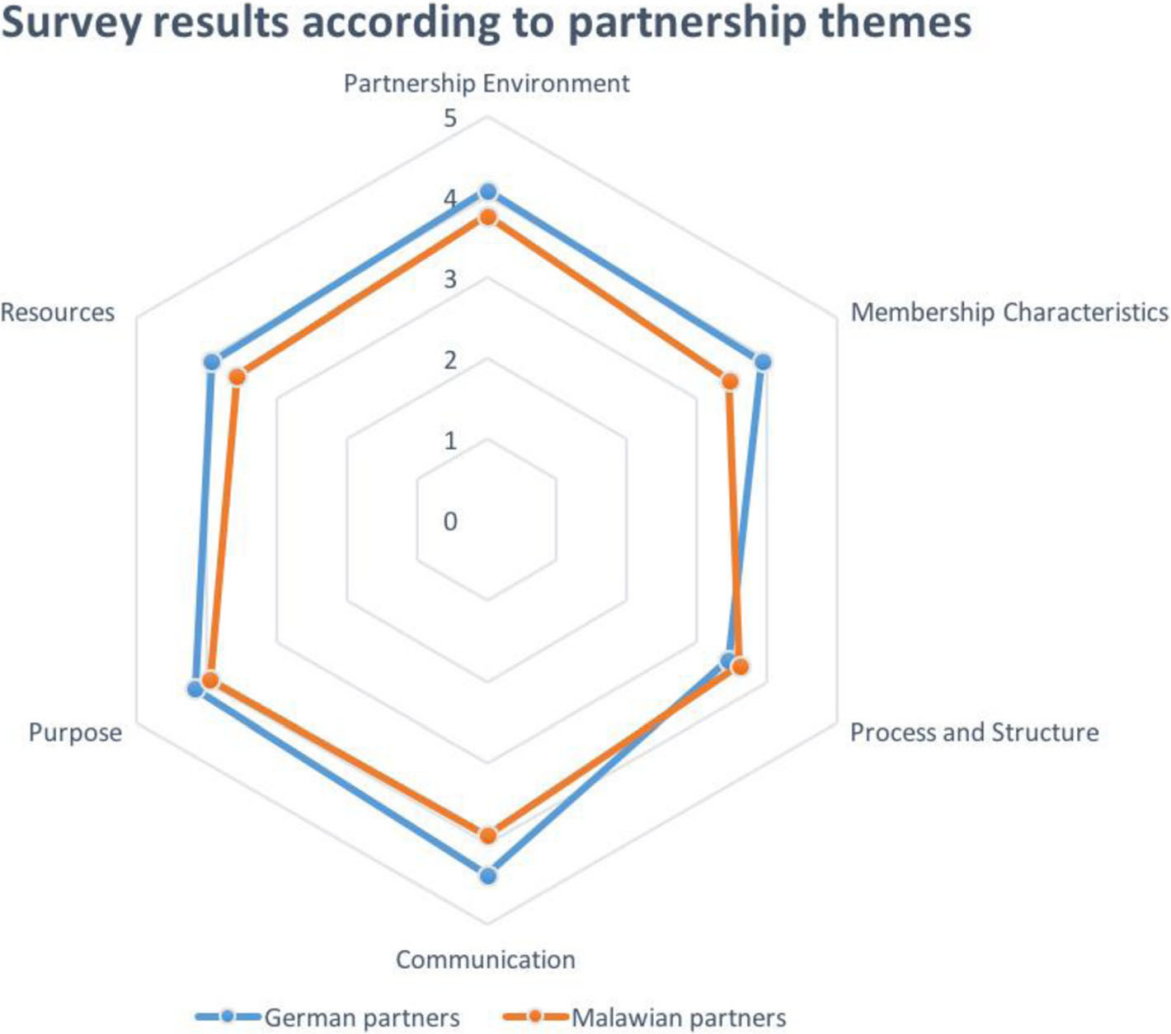
Results of survey showing responses by Malawian and German partners separately, whereby each spoke represents one of the six overall themes of the partnership evaluation as on the Likert-scale from 1 to 5 (1 = no agreement, 5 = high agreement): Partnership Environment, Membership Characteristics, Processes and Structure, Communication, Purpose and Resources

**Fig. 2:**
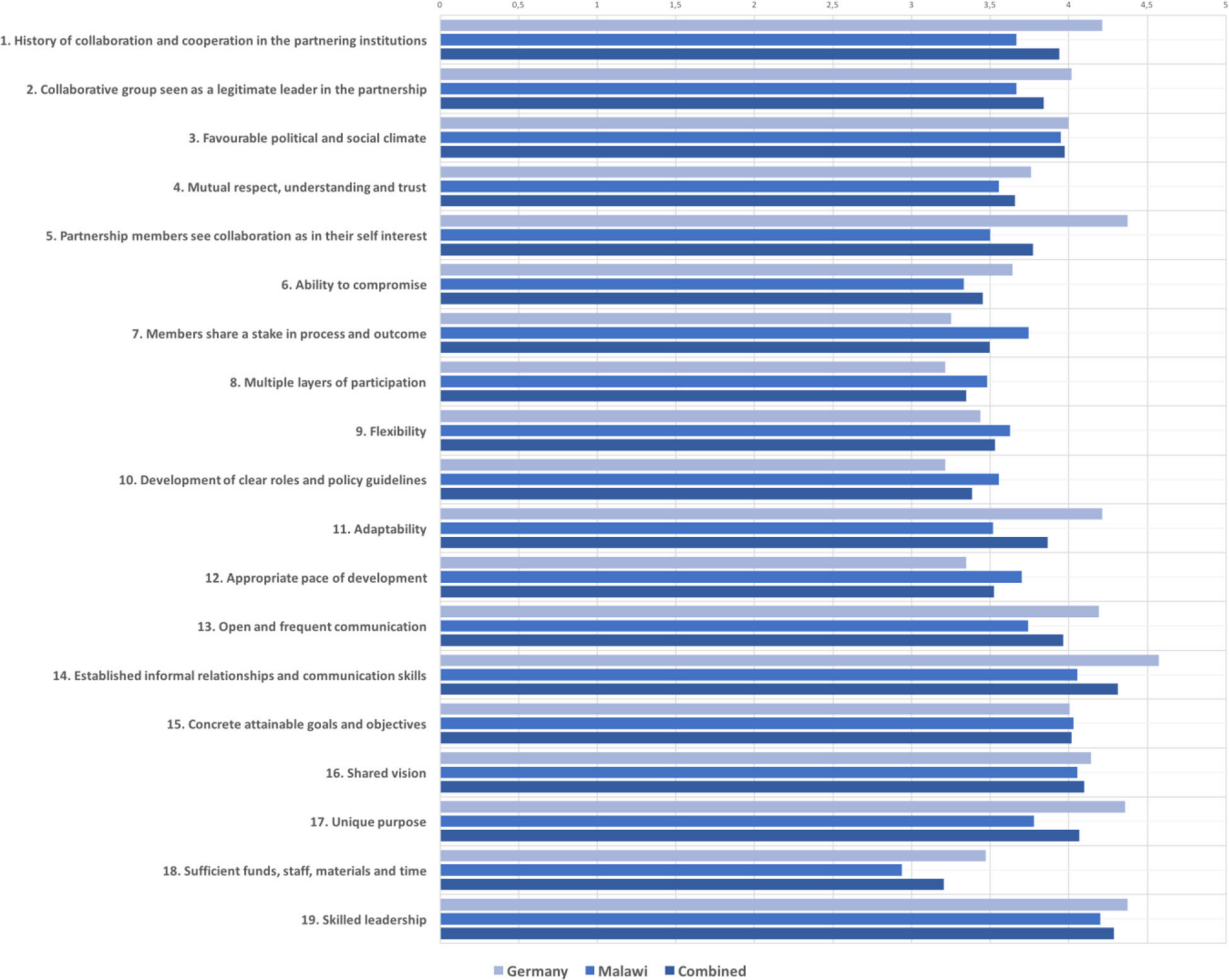
Means of responses to individual factors by country of responders which are part of six overall themes: Partnership Environment, Membership Characteristics, Processes and Structure, Communication, Purpose and Resources. Likert scale from 1 to 5 (1 = no agreement, 5 = high agreement)

The factor scoring lowest by partners was for insufficiency of funds, staff, materials and time. Respondents saw too little compromise within partnership decisions. The overall process and structure of the partnership was rated poorly. According to the survey, members of the partnership had insufficient stake in processes and outcomes; the participation was unequal and lacked flexibility. There was no clear development of partnership roles and policy guidelines, and the development pace was not seen as appropriate. There was also a discrepancy in scoring between Malawian and German respondents. German respondents’ ratings showed a wider range of scores compared to Malawians (Figs. 1 and 2).

## Discussion

The ESTHER-MAGNET partnership between the KCH Medical Department and a consortium of initially three - later two - German university departments led by the Institute of Public Health Heidelberg lasted from 2008 to 2015, and covered three funding periods. The partnership focused on capacity strengthening in clinical care, teaching and mentoring of intern doctors and conducting operations research. Throughout all project phases, the overall partnership objective remained, but specific foci changed according to mutually agreed upon topics endorsed by the funding agent. The strength of the partnership was rooted in a solid knowledge of local needs, in-situ cooperation, capacity to adapt solutions and flexibly within a changing partnership environment, flat hierarchies, and a strong focus on individual and institutional human capacity development also serving as an incentive for local partners that were given, for example, the possibility for external trainings.

Over a period of eight years, MAGNET has undoubtedly made a considerable contribution to patient care, teaching and capacity strengthening at the KCH. Considering the limited resources available (human, financial and infrastructural), the partnership generated significant outputs, but eventually failed to produce self-sustaining structures and processes to withstand multiple changes and challenges.

### Survey

The results of the survey have to be viewed against the conflicts towards the end of the partnership and the underrepresentation of partners involved in the earlier phases. Nevertheless, the discrepancies in judgement as well as the concurrences underline that frictions had developed by the end of the partnership: for example, low scores for the categories of *mutual respect* and the *ability to compromise*, contrasted the original intentions of the partnership agreement. The low rating for *sufficient funds, staff, materials and time* is more of a general concern than a condition specifically attributable to the partnership. All available funds were supplemental to the general hospital budget and allowed for additional activities, equipment and supplies.

### Strategy

Cooperation within a partnership is only successful if the partners agree on a common strategy to achieve the goals. Within the CW model, a strategy is defined as a pattern within a decision stream. The pattern can only develop if the partners mutually negotiate one or more common objectives [24].

Although based on a mutually agreed upon strategy, the degree to which the MAGNET partners followed the strategic pathway fluctuated. Problems addressed by the partnership frequently reached the limits of departmental control, for example, for the number of staff and supplies. The analysis of the MAGNET healthcare quality improvement process revealed that it was “essential to identify modifiable factors that are under the control of the Medical Department” so the staff would not be overwhelmed by tasks that constituted “a huge challenge” [20]. Even within these limits, considerable improvement can be achieved provided staff is sufficiently motivated [16]. Staff motivation is pivotal, but influenced by a number of factors described by Franco et al. [36]: “… the individual, the immediate organizational work context, and the cultural context”. The partnership strategy aimed to stimulate health workers’ motivation, but this eventually failed because of other overriding influences of organizational factors and health sector-related problems [36].

### Cooperation

The basis for a good cooperation as defined in the CW model includes confidence, negotiation of appropriate forms of cooperation, and transparency of partnership roles. A partnership creates a new social system from common goals, involved stakeholders, stakeholder relationships, and partnership rules [24]. A predominant factor for the success and failure of a partnership is mutual trust, and the willingness to accomplish agreed upon objectives despite obvious challenges. The lack of continuity of Malawian partners had a significant impact on this partnership. When the founding Malawian partners left KCH, an imbalance of knowledge and sense of ownership among the subsequent local partners was created. Changes in staff maybe more likely to occur in central teaching hospitals, since staff at all levels may be promoted. Changes at the level of hospital administration can also be triggered in post-election periods or programmatic changes. MAGNET was affected in particular by the unusual repeated changes in the HoD. The potential effect of these changes has to be considered in partnerships wherein agreements are less dependent on specific individual partners.

The increasing influence of the funding agency over time partially changed the perception of the partnership as being donor-driven. This can be exemplified by the low rating for *Sufficient funds, staff, materials and time.* Both German and Malawian partners perceived that the MAGNET’s objectives were more determined by an external third party rather than mutually agreed goals among the hospital partnership. Likewise, the external review assessed the partnership as an implementation project and not as a partnership handled by the involved partners.

### Programme steering

Agreements between partners are the basis for mutually preparing and making relevant decisions and providing program steering. Steering provides the structure for cooperation to make strategic and operational decisions, business and resource management, operational planning, and implementation and monitoring. Within the CW model, steering defines the rules, roles, mandates and responsibilities in the decision-making process [24]. In the first phase of MAGNET, the committed and strong Malawian and German leadership had a dominant role for steering the project, but lost power in the subsequent phases. The external evaluation illustrated conflict between an independent partnership and the project funded by a donor, but implemented by the partnership. The partnership had to adapt to a changing departmental environment, whereas the funding agent wanted to see a project implemented with a time-bound working plan. Both efforts can only partially co-exist. On the one hand, the funder had signed a contract with only the German institution, but on the other hand requested a country-led project management.

Decisions by other powerful actors in the health sector had an enormous impact on partnership efforts, as illustrated by the MoH decision to set up a dialysis unit in the Medical Department or the opening of the Lilongwe campus of the CoM. Whether these decisions were favourable for the hospital or health care in general is not the question, but they did create a difficult situation with conflicting objectives for the department to which the partnership had to adjust. Eventually, the partnership structures and its role and influence were too weak to survive the multiple changes in institutional development, interpersonal relationships and donor policies.

### Processes

In a successful cooperation, effective forms of service are clearly defined, as are the ways that new processes are established or existing processes are adapted within the partnership. Challenges for establishing and adapting process are handled jointly through cooperation within the partnership [24]. It was notable that both the document review and survey concurred in the low rating of partnership processes and structures. Despite the partnership’s efforts and intentions, robust, self-sustaining processes could not be implemented. This judgement was supported by the results of the quality analysis performed at the department that identified demonstrated weak internal processes [20]. There are several contributing factors, such as the limited human resources, lack of staff continuity on the Malawian side, fewer intense personal contacts, KCH’s structural weakness in terms of self-management and decision-making power, as well as unpredictable budgets. Due to the abrupt ending of the partnership we cannot conclusively state which processes are still in place.

### Learning and innovation

The CW model advises that partnerships should provide a constructive environment for innovation that is fostered by strengthening learning competencies of involved actors and by adapting rules, structures, processes and rituals accordingly [24]. This was partially addressed in MAGNET through the operations research and the annual departmental meeting.

Edwards [1] describes four types of health partnerships depending on the focus of capacity building: individual versus organisational capacity and generic versus specialist skills. We believe the focus of the MAGNET partnership was on the organisation rather than individuals [1], although individuals did benefit. The strategy on capacity strengthening was set on education and training, rather than specialist building. In Edwards’ review, this approach was associated with higher sustainability. The contribution of MAGNET to sustained quality improvement is difficult to judge at this time. MAGNET certainly has stimulated processes for mentoring interns. However, there is a need to develop appropriate tools for continuous partnership evaluation based on agreed upon principles and specifically adapted to the scope of the particular partnership [37].

Partnerships like MAGNET stress issues of appropriate structure and function of a central teaching hospital at a tertiary care level that are not yet answered for Malawi. What kind of care and to which level of sub-specialization should and can care be provided at KCH? What are the priorities for care if resources are limited? What is the right ratio between in- and out-patient care? How can treatment, needs of care and teaching be optimally co-organised?

## Conclusion

The presented evaluation of the MAGNET hospital partnership and its abrupt ending after a long period of cooperation, contributes to the discussion of the role and contribution of health partnerships in achieving global health objectives. Towards the end of the partnership, the partners failed to communicate and thus lost a shared perspective on objectives and expectations. Comparisons of partnerships are difficult [1], hence we are cautious about generalising our observations.

Funding agents should understand specific partnership characteristics and allow for autonomy rather than exploiting partners for their own agenda. We caution about overloading local partnerships with high expectations based on a global agenda. The history of the MAGNET partnership also demonstrates the rich potential of an international hospital partnership approach to improve and drive change in health care delivery. Partnerships offer a significant opportunity to respond and adapt to needs and change much faster than donor agencies can. Nevertheless, partnerships should stay aligned with national programmes and remain in dialogue with the development agencies, especially with regard to partners’ experiences. Donor agencies and MoHs should utilize experiences generated by health partnerships.

## Abbreviations

CIM: Centre for international migration and development
CME: Continuous medical education
CoM: College of medicine
ESTHER: Ensemble pour une Solidarité Thérapeutique Hospitalière En Réseau
GIZ: German international cooperation
HoD: Head of department
KCH: Kamuzu central hospital
MAGNET: Malawi German networking for capacity building in treatment, training and research
MD: Medical department (in the context of this paper: at the KCH)
MMed: Master of medicine
MoH: Ministry of health
